# Transcriptome responses of RNAi-mediated *ETH* knockdown in *Scylla paramamosain* at different premolt substages

**DOI:** 10.3389/fendo.2022.917088

**Published:** 2022-07-28

**Authors:** Siuming-Francis Chan, Qi-Qiao Wen, Chun-Mei Ao, Wei Wang, Cheng-Gui Wang, Yan-Fei Zhao

**Affiliations:** ^1^ College of Fisheries, Guangdong Ocean University, Zhanjiang, China; ^2^ Guangxi Key Laboratory of Beibu Gulf Marine Biodiversity Conservation, Beibu Gulf University, Qinzhou, China

**Keywords:** ecdysis triggering hormone, RNA interference, transcriptome, molt, downstream, *Scylla paramamosain*

## Abstract

Ecdysis triggering hormone (ETH) plays an important role in molting, reproduction, and courtship behavior in insects. To investigate the potential downstream pathways and genes of ETH in *Scylla paramamosain*, RNA interference (RNAi) was conducted on crabs at early (D0) and late (D2) premolt substages, and the transcriptome profiles of each group were compared by RNA sequencing. Real-time quantitative polymerase chain reaction (RT-qPCR) and semiquantitative polymerase chain reaction (RT-PCR) results showed a significant knockdown of *ETH* at D0 stage, whereas a significant increase was shown conversely in crabs at D2 substage after the injection of *dsETH*. A total of 242,979 transcripts were assembled, and 44,012 unigenes were identified. Transcriptomic comparison between crabs at D2 and D0 substages showed 2,683 differentially expressed genes (DEGs); these genes were enriched in ribosome and pathways related to transcription factor complex and cell part. Twenty DEGs were identified between *dsETH*-injected and *dsGFP*-injected crabs at D0 substage; these DEGs were involved in carbohydrate metabolism, one carbon pool by folate, and chitin binding. Twenty-six DEGs were identified between *dsETH*-injected and *dsGFP*-injected crabs at D2 substage; these DEGs were involved in calcium channel inhibitor activity, fat digestion and absorption, and cardiac muscle contraction. RT-qPCR verified the differential expression of the selected genes. In conclusion, crabs at D0 substage are more active in preparing the macromolecular complex that is needed for molting. Moreover, ETH has potential roles in carbohydrate metabolism, one carbon pool by folate, and chitin binding for crabs at D0 substage, while the role of ETH turns to be involved in calcium channel inhibitor activity, fat digestion and absorption, and cardiac muscle contraction at D2 substage to facilitate the occurrence of molting. The selected DEGs provide valuable insight into the role of ETH in the regulation of crustacean molting.

## Introduction

Molting is a natural and biological process for survival, development, and reproduction in arthropods. The molting cycle can be divided into four stages: intermolt stage (C), premolt stage (D), ecdysis (E), and postmolt stage (A, B) (1). In crustaceans, the premolt stage can be divided into five substages: early (stage D0), mid (stage D1), and late (stages D2, D3, D4) substages (2). During the premolt stage, the old cuticle detaches and the inner layers of the old cuticle are degraded and reabsorbed, the new exoskeleton is synthesized, the claw closer muscle atrophies, and autotomized appendages regenerate (3–5). The molting process is achieved by the interaction of ecdysteroids and a complex suite of interacting neuropeptides (6). As an essential hormone in crustaceans, 20-hydroxyecdysone (20E) fluctuates with the process of molting (7) and is controlled by neuropeptides secreted from the central nervous system and then regulates a series of target genes through ecdysone receptor signaling (8, 9).

As one of the target genes, *ecdysis triggering hormone* (*ETH*) has an ecdysone response element in its promoter region. Also, Gauthier etal. (10) showed that the *Drosophila* homolog of ATF4 (cryptocephal) is an ecdysone receptor coactivator. The ecdysone receptor can bind to the promoter region of *ETH* with cryptocephal and initiates the transcription of *ETH*. Researchers have found that ETH has additional roles besides initiating ecdysis. In insects, ETH plays dual roles, initiation of ecdysis and trachea clearance (11, 12). In *Drosophila*, the knockdown of *ecdysis triggering hormone receptor* (*ETHR*) revealed differential expression of genes involved in axon guidance, courtship behavior, and chemosensory functions, which indicated a role in the regulation of courtship behavior (13). Researchers also found that ETH acts as a regulatory peptide to ensure the synthesis of juvenile hormone at the proper time (14). As a dual regulator of octopamine and juvenile hormone release, ETH also provides a link between the stress-induced elevation of ecdysone levels and consequent reduction in fecundity in *Drosophila* (15). ETH in *Bactrocera dorsalis* also plays an essential role in its reproduction, *via* the regulation of JH and vitellogenin, and is controlled by a pulse of 20E (16). ETH also regulates molting in crustaceans (17). In *Scylla paramamosain*, we found that the knockdown of *ETH* leads to failure of molting at the early premolt substage. Interestingly, the transcript level of *ETH* had a significant increase conversely when crabs at the late premolt substage (D2) were injected with the same amount of *dsETH* as at the early premolt stage (D0), which suggests different means of regulation of *ETH* at the D0 and D2 substages in *S. paramamosain* (18).

In crustaceans, the premolt stage is essential for adequate preparation for molting. Many biological processes are occurring at the premolt stage, amounts of them such as the regulation of apolysis have not been well-studied. Transcriptome analyses have mostly focused on the regulatory changes among A/B, C, and D molt stages. However, analyses between the different substages of the premolt stage in crustaceans are rare despite their potential to reveal the dynamic changes in transcriptome in the preparation for molting. Although the roles of ETH are well-studied in insects, related work is still limited in crustaceans, and whether ETH has different roles in crustaceans is still unknown. Notably, ETH can regulate the initiation of ecdysis in crustaceans (17); however, its downstream transcriptional pathways remain largely elusive. In this study, *S. paramamosain* was selected. To reveal the dynamic change of biological processes at the premolt stage and determine the roles of ETH at the D0 and D2 substages in *S. paramamosain*, RNAi was used. This study aimed to identify candidate genes and pathways related to ETH and determine the role of ETH at the D0 and D2 substages.

## Materials and methods

### Animals

Crabs were collected from the coastal area of Zhanjiang, Guangdong Province, China. Individuals (i.e., 2.4–2.9 g) were selected and cultured in 1-m^3^ concrete ponds with seawater at 24°C–27°C and 20‰ salinity and fed daily with oysters. After a week, healthy crabs at the D0 and D2 substages (19) were selected for the RNAi experiment.

### RNAi and injection

Primers flanked by the T7 promoter sequence were designed and used to synthesize the double-stranded RNA (dsRNA) for *ETH* and control gene (*GFP*) (18). The dsRNA templates were amplified with the procedure: 95°C for 5 min; followed by 30 cycles of 95°C for 30 s, 57°C for 30 s, and 72°C for 30 s; then 72°C for 10 min. Then, PCR products were purified using FastPure Gel DNA Extraction Mini Kit (Vazyme, Nanjing, China). dsRNA was produced with the above DNA templates under the method of T7 RiboMAX™ Express RNAi Synthesis kit (Promega, Beijing, China). The final dsRNA was diluted to 1 μg/μl. Crabs (n = 20) at the D0 and D2 substages were collected and separately injected with *dsETH* and *dsGFP*. Each crab was injected in dsRNA at a dose of 5 μg/g. After 48 h, thoracic ganglia were sampled and analyzed, each sample was collected from five crabs, and each group has three samples.

### RT-PCR

According to the target genetic sequence, specific primers were designed using Primer 5.0 and 18s rRNA was used as the internal reference gene (**Table S1** **)**. For sequence amplification, the following program was used: 94°C for 5 min followed by 30 cycles of 94°C for 30 s, 57°C for 30 s, 72°C for 30 s, and 72°C for 10 min. RT-PCR reactions of 20 μl contained 1 μl of cDNA and each primer, 10 μl of 2× PCR Master Mix (GenStar, Beijing, China), and 7 μl ddH_2_O. Amplified products were electrophoresed on 1% agarose gel and imaged using ImageJ 1.51j8 software performs data conversion.

### Tissue collection, RNA isolation, and cDNA library preparation

Crabs were chilled on ice and dissected for thoracic ganglia. For every group, samples were collected in three different pools; each pool contained five biological replicates. Total RNA was prepared using a column-based TransZol Up Plus RNA kit (TransGen Biotech, China). The concentration of the extractive RNA was determined using the Thermo Fisher Scientific Nanodrop 2000 (Waltham, MA, USA), and the quality and integrity were determined with an Agilent 2100 Bioanalyzer (Agilent, Palo Alto, CA, USA) and a Qubit 2.0 Fluorometer (Life Technologies, CA, USA). The qualified RNA samples were used for cDNA library construction. Sequencing was done with the NEBNext R Ultra™ RNA Library Prep Kit for Illumina R (NEB, Ipswich, MA, USA), and index codes were added to tag these sequences. The libraries were sequenced using the Illumina HiSeq platform.

### Cleaning, *de novo* assembly, and annotation of sequencing reads

Raw reads were cleaned by removing adapter, primer sequences, reads with ambiguous nucleotides larger than 5%, and low-quality reads. *De novo* assembly was done using Trinity (20). For annotation, BLASTX searches were performed with e-value <1e-5 and 1e-3 against the NR (NCBI nonredundant protein sequences), NT (NCBI nonredundant nucleotide sequences), KO [Kyoto Encyclopedia of Genes and Genomes (KEGG) ortholog database], Swiss-Prot (a manually annotated and reviewed protein sequence database), Pfam (protein family), GO (Gene Ontology) databases, and KOG/COG (clusters of orthologous groups of proteins). The read count of every gene with FPKM based on the length and read count was calculated. The raw sequencing reads are archived at the NCBI Sequence Read Archive: PRJNA813292.

### Differentially expressed gene analysis

Differential expression analyses of D2dsGFP vs. D0dsGFP, D0dsETH vs. D0dsGFP, and D2dsETH vs. D2dsGFP were performed using DESeq2 software (21). Unigene with false discovery rate (padj) values <0.05 and |log2(FoldChange)| >1 were set as the threshold for differentially expressed genes (DEGs). GO and KEGG database annotations were further analyzed (corrected *P*-value <0.05) to explore the functions of DEGs in *S. paramamosain*.

### Validation with RT-qPCR

To analyze the knockdown efficiency of *ETH* and validate the DEGs from the transcriptome data, RT-qPCR reactions were performed with the RT-qPCR machines (Bio-Rad CFX Connect PCR, Bio-Rad, USA). Primers were designed using the Software Primer 5.0, and 18s rRNA was used as internal control genes (**Table S1**). RT-qPCR reaction system: 10 μl of SYBR qPCR Master Mix (Vazyme, Nanjing, China), 2 μl of cDNA, 7 μl of ultrapure water, and 0.5 μl of each Forward and Reverse primer (10 μM). RT-qPCR reaction condition: 95°C for 2 min, followed by 39 cycles of 95°C for 5 s and 55°C for 30 s, and then a melt curve analysis was performed. The relative mRNA abundance of each gene was calculated by the ΔΔCt method, then normalized by that of 18S for each sample. The reaction was performed in triplicate for each sample, and five mud crabs were analyzed in each group. The relative expression of the target genes between groups was statistically analyzed with GraphPad Prism 8.0.1 (GraphPad Software Inc., San Diego, CA, USA) by one-way ANOVA, followed by Tukey’s honestly significant difference test and Pearson correlation coefficients. All data were expressed as means ± standard deviation (SD).

## Results

### Knockdown of *ETH* before transcriptomic analysis

**Figure 1:**
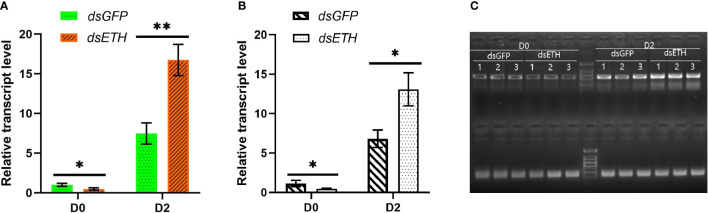
Relative transcript levels of *ETH* in thoracic ganglia of *dsGFP-* and *dsETH*-injected crabs at the D0 and D2 substages were measured respectively [by Student’s t-test, values are means ± SD (n = 3), *P < 0.05; **P < 0.01]. **(A)** RT-qPCR result; **(B)** RT-PCR result; **(C)** Images of the gels for RT-PCR.

Both RT-qPCR and RT-PCR results showed that the transcript level of *ETH* was decreased significantly (*P* < 0.05) after the injection of *dsETH* at the D0 substage, whereas the transcript level of *ETH* had a significant increase conversely when crabs at the D2 substage were injected with the same amount of *dsETH* (**Figure 1**).

### Sequencing and *de novo* transcriptome assembly and annotation

From 12 thoracic ganglia samples of the above four groups, a total of 252,013,587 clean reads from 267,917,841 raw reads were obtained after sequencing, quality trimming, and adapter clipping. None of the error rates of the 12 libraries exceeded 0.03%, and the mean Q20 and Q30 were 97.51% and 93.57%. After *de novo* assembly, 242,979 transcripts and 124,470 unigenes were obtained, which had mean lengths of 1,326 bp and 1,006 bp (**Table 1**). The length distributions of all the transcripts and unigenes are shown in **Figure S1**. The annotation results are shown in **Table 1**.

**Table 1:** Summary of sequence analysis in *S. paramamosain* thoracic ganglia.

		Number	Percentage (%)
**Sequencing**	Raw reads	267,917,841	
	Clean reads	252,013,587	
	Mean of Q20		97.51
	Mean of Q30		93.57
**Assembly**	Number of transcripts	242,979	
	Number of unigenes	124,470	
	Mean length (nt) of transcripts	1,326	
	Mean length (nt) of unigenes	1,006	
**Annotation**	Annotated in NR	22,691	18.23
	Annotated in NT	10,418	8.36
	Annotated in KO	7,989	6.41
	Annotated in SwissProt	12,323	9.9
	Annotated in PFAM	31,687	25.45
	Annotated in GO	31,679	25.45
	Annotated in KOG	6,974	5.6
	Annotated in all Databases	2,588	2.07
	Annotated in at least one Database	44,012	35.35

### Differentially expressed gene identification

**Figure 2:**
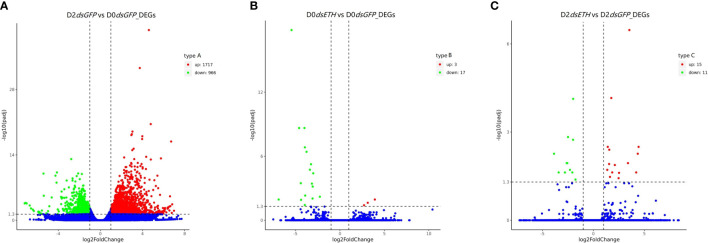
Differentially expressed genes (DEGs) in three comparisons of *S. paramamosain* [corrected *P*-value <0.05 and |log2(fold change)| >1]. **(A)** Volcano plot of DEGs in D2dsGFP (Crabs post dsGFP injection at D2 substage) vs. D0dsGFP (Crabs post dsGFP injection at D0 substage). **(B)** Volcano plot of DEGs in D0dsETH vs. D0dsGFP. **(C)** Volcano plot of DEGs in D2dsETH vs. D2dsGFP. Red spots represent upregulated genes and green spots indicate downregulated genes. Blue spots represent genes showing no obvious change in the comparison.

We found in total 2,708 DEGs in the transcriptomes using the corrected *P*-value <0.05 and |log2(fold change)| >1 (**Figure 2**, **Table S2**). The DEGs between D2*dsGFP* and D0*dsGFP* were 1,717 upregulated genes and 966 downregulated genes (**Figure 2A**; **Figure S2**). The DEGs were few in D0*dsETH* vs. D0*dsGFP*, with only 3 upregulated genes and 17 downregulated genes (**Figure 2B**; **Figure S3**); 15 upregulated genes and 11 downregulated genes were found in D2*dsETH* vs. D2*dsGFP* (**Figure 2C**; **Figure S4**).

### Enrichment analysis and functional classification of differentially expressed genes

**Figure 3:**
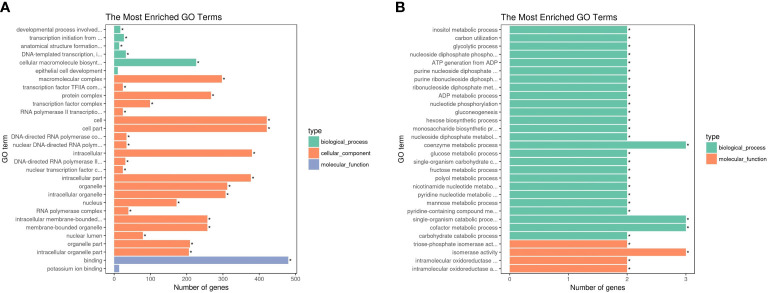
Enrichment of differentially expressed genes (DEGs) in GO (corrected *P*-value <0.05). **(A)** Downregulated DEGs that enriched GO terms in D2*dsGFP* vs. D0*dsGFP*. **(B)** Downregulated DEGs that enriched GO terms in D0*dsETH* vs. D0*dsGFP*. Significant difference compared with control indicated by an asterisk (P-value <0.05).

**Figure 4:**
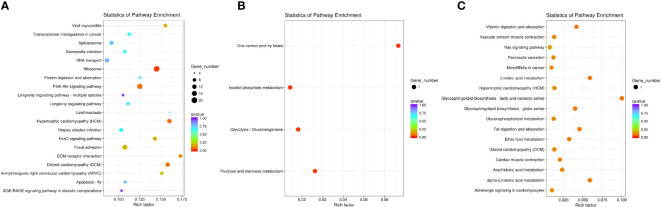
Enrichment of differentially expressed genes (DEGs) in KEGG (corrected *P*-value <0.05). **(A)** Downregulated DEGs that enriched KEGG terms in D2*dsGFP* vs. *D0dsGFP*. **(B)** Downregulated DEGs that enriched KEGG terms in D0*dsETH* vs. D0*dsGFP*. **(C)** Upregulated DEGs that enriched KEGG terms in D2*dsETH* vs. D2*dsGFP*.

**Figure 5:**
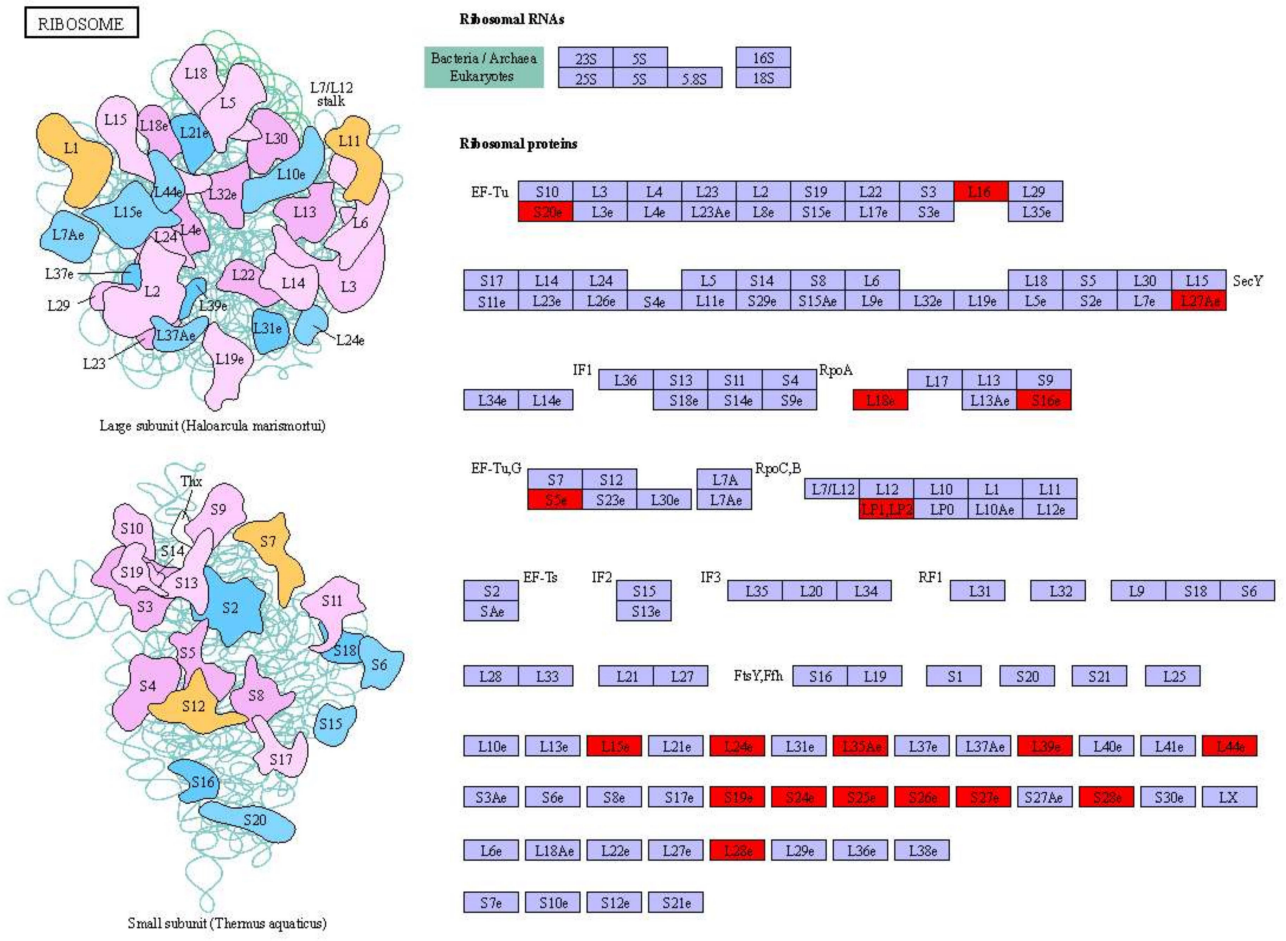
Significantly differentially expressed genes of ribosome-related pathways in D2*dsGFP* vs. D0*dsGFP* of *S. paramamosain*. Red indicates significantly upregulated genes. (For interpretation of the short letters’ meaning in this figure legend, the reader is referred to the web version of KEGG: ko03010.).

GO and KEGG pathway analyses of DEGs were performed (corrected *P*-value <0.05) to determine their functions in *S. paramamosain*. DEGs between D2*dsGFP* and D0*dsGFP* were annotated (padj <0.05). Enrichment was not observed in either GO terms or KEGG pathways for upregulated DEGs. Compared to the D0*dsGFP* group, for downregulated DEGs, GO enrichment revealed that transcription factor complexes such as macromolecular complex, transcription factor complex, and RNA polymerase II transcription factor complex were the most enriched subcategories in the cellular component. In addition, cell part (means any constituent part of a cell, the basic structural and functional unit of all organisms), bind, and intracellular organelle part were enriched (**Figure 3A**; **Table S3**). KEGG results of downregulated DEGs were mainly enriched in ribosome (**Figures 4A**, **5** **;** **Table S4**).

GO enrichment revealed that the downregulated DEGs of the D0*dsETH* group involved in the biological process were mainly enriched in energy metabolic processes such as inositol metabolic, glycolytic process, mannose and fructose metabolic, and adenosine triphosphate (ATP) generation from adenosine diphosphate (ADP). In the molecular function category, downregulated DEGs were mostly enriched in catalytic activity and binding such as triosephosphate isomerase activity and intramolecular oxidoreductase activity (**Figure 3B**; **Table S3**). Upregulated DEGs of the D0*dsETH* group were enriched in chitin binding (**Table S3**). For KEGG pathways, downregulated DEGs of the D0*dsETH* group were enriched in one carbon pool by folate, fructose and mannose metabolism, glycolysis/gluconeogenesis, and inositol phosphate metabolism, which were related to the energy metabolic pathway (**Figure 4B**; **Table S4**).

Enrichment was not observed in either GO terms or KEGG pathways for DEGs between the D2*dsETH* and D2*dsGFP* groups. To be as inclusive as possible, less strict screening criteria (*P*-value <0.05 instead of corrected *P*-value <0.05) were applied, and upregulated DEGs of the D2*dsETH* group were mainly enriched in the regulation of cell shape, regulation of Wnt signaling pathway, globoside metabolic process, alpha-(1,2)-fucosyltransferase activity, and regulation of cell morphogenesis under GO enrichment; downregulated DEGs were mainly enriched in calcium channel inhibitor activity, endopeptidase inhibitor activity, and defense response (**Table S3**). KEGG enrichment results show that upregulated DEGs of the D2*dsETH* group are involved in glycosphingolipid biosynthesis, linoleic acid metabolism, fat digestion and absorption, vitamin digestion and absorption, ether lipid metabolism, and arachidonic acid metabolism; downregulated DEGs of the D2*dsETH* group were not enriched in the KEGG pathway (**Figure 4C**; **Table S4**).

### Differentially expressed genes involved in carbohydrate metabolism, lipid metabolism, and biological process of molting

**Figure 6:**
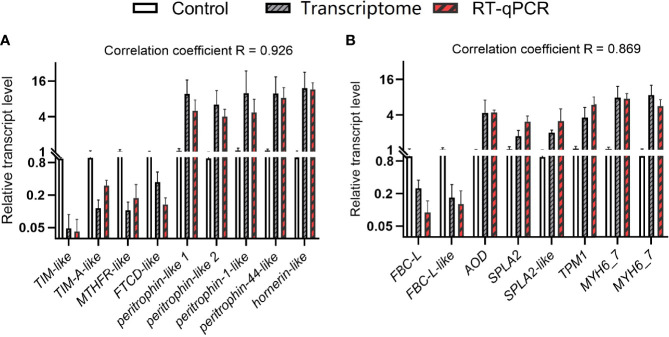
Comparison of gene expression patterns obtained by RNA-Seq and RT-qPCR. Values are means ± SD (n = 3). **(A)** D0*dsETH* vs. D0*dsGFP*. **(B)** D2*dsETH* vs. D2*dsGFP*.

Besides the analysis of the entire gene set, we specifically checked for genes that were significantly regulated and involved in carbohydrate metabolism, one carbon pool by folate, chitin binding, calcium channel inhibitor activity, fat digestion and absorption, and cardiac muscle contraction (**Table 2**). At the D0 substage, the knockdown of *ETH* caused the downregulation of *triosephosphate isomerase-like* (*TIM-like*) and *triosephosphate isomerase A-like* (*TIM-A-like*), genes related to glycolytic enzyme that are essential to carbohydrate metabolism. Also, a gene of *methylenetetrahydrofolate reductase-like* (*MTHFR-like*) was downregulated, which was a critical enzyme of the folate pathway and required for DNA synthesis, methylation, and amino acid metabolism. Whereas a unigene related to chitin binding (*peritrophin-like1*) was upregulated significantly. At the D2 substage, the knockdown of *ETH* showed the downregulation of *fibrocystin-L* that was related to calcium channel inhibitor activity and essential to cellular control of adhesion and epithelial morphogenesis (22), whereas *amine oxidase* (*AO*) related to fat digestion and absorption and the unigene of *tropomyosin 1* (*FBC-L*) that participates in cardiac muscle contraction were upregulated. To confirm the reliability of these transcriptome data, these selected DEGs were determined with RT-qPCR (**Figure 6**). The correlation coefficient R was 0.926 and 0.869.

**Table:** **Table 2** DEGs related to metabolism and molting process from *S. paramamosain* responding to *dsETH* treatment at different premolt substages.

	Unigene name	Up/down	Log_2_FoldChange	pval	padj	Description(blast)	Length (bp)
CD0*dsETH* vs. D0*dsGFP*	**Carbohydrate metabolism (ko00010, ko00051, ko00562)**						
	*TIM-like*	Down	-4.3993	2.53E-08	0.00033934	triosephosphate isomerase-like	1115
	*TIM-A-like*	Down	-3.251	2.82E-10	5.6559E-06	triosephosphate isomerase A-like	2392
	**One carbon pool by folate (ko00670)**						
	*MTHFR-like*	Down	-3.3763	1.07E-09	1.84E-05	methylenetetrahydrofolate reductase-like	5300
	*FTCD-like*	Down	-1.5911	0.016123	0.99796	formimidoyltransferase-cyclodeaminase-like	2011
	**Chitin binding (GO:0008061)**						
	*Peritrophin-like 1*	Up	3.1302	3.43E-06	0.022955	peritrophin-like 1	1144
	*Peritrophin-like 2*	Up	2.5507	0.000111	0.23835	peritrophin-like 2	1182
	*Peritrophin-1-like*	Up	3.2315	0.000831	0.73007	peritrophin-1-like	407
	*Peritrophin-44-like*	Up	3.1439	0.000366	0.47854	peritrophin-44-like	1848
	*Hornerin-like*	Up	3.4476	6.59E-05	0.18041	hornerin-like	1925
D2*dsETH* vs. D2*dsGFP*	**Calcium channel inhibitor activity (GO:0019855)**						
	*FBC-L*	Down	-1.9933	7.47E-08	0.0018296	fibrocystin-L	6692
	*FBC-L-like*	Down	-2.5596	8.42E-05	0.23983	fibrocystin-L-like-partial	1105
	**Fat digestion and absorption (ko04975)**						
	*AOD*	Up	2.1034	1.62E-06	0.013212	amine oxidase	1624
	*SPLA2*	Up	0.79017	0.014509	1	secretory phospholipase A2	2181
	*SPLA2-like*	Up	1.0174	0.006331	1	secretory phospholipase A2-like	862
	**Cardiac muscle contraction (ko04260)**						
	*TPM1*	Up	1.8475	3.98E-06	0.023608	tropomyosin 1	650
	*MYH6*	Up	2.9451	0.000408	0.59106	myosin heavy chain 6	5228
	*MYH7*	Up	3.0999	0.000153	0.36105	myosin heavy chain 7	5092

To be as inclusive as possible, less strict screening criteria of DEGs (pval <0.05 instead of padj <0.05) were applied. From these DEGs, *formimidoyltransferase-cyclodeaminase-like* (*FTCD-like*), a folate-dependent enzyme that displays both transferase and deaminase activity (23), was selected and downregulated with the knockdown of *ETH* at the D0 substage. Also, another four genes (*peritrophin-like 2*, *peritrophin-1-like*, *peritrophin-44-like*, and *hornerin-like*) involved in chitin binding were upregulated. At the D2 substage, *fibrocystin-L-like-partial*, involved in calcium channel inhibitor activity, was downregulated with the knockdown of *ETH*, two genes (*secretory phospholipase A2*, *secretory phospholipase A2-like*) related to fat digestion and absorption and two genes (*myosin heavy chain 6/7*) related to cardiac muscle contraction were upregulated with the knockdown of *ETH*. These DEGs were also verified using RT-qPCR, and the result showed high consistency with transcriptome sequencing.

## Discussion

In the previous study, the expression level of ETH showed a periodic fluctuation with molting, and ETH exists not only at the E stage but also at the other stages (18). Functional diversity of ETH has been proven in insects (not just triggering ecdysis and working at the E stage) (14–16). The existence of ETH at other stages meant that it may focus on triggering not only ecdysis but also other aspects. When we injected *dsETH* to crabs at the D0 substage, the RT-sqPCR result showed a trend: decline. It meant that we knocked down the ETH successfully. Meanwhile, RT-sqPCR results showed the increased trend at the D2 substage. It might be due to the later retaliatory rise of the transcriptive level offsetting the knockdown at an early time after the injection of *dsETH*. To balance, the injection of *dsETH* at the D2 substage led to an increase of *ETH* when we did sampling for transcriptome analysis. Our previous study showed that ETH was regulated by different models in *S. paramamosain*, and eclosion hormone was important to the regulation of *ETH* at the D2 substage (18).

In arthropods, apolysis is considered as an initial sign of molting and triggered by rising 20E titer (24, 25). Immediately after apolysis, the molting fluid is secreted into the new apolysial space and initiates a series of biological processes such as new cuticle secretion, old cuticle degradation, and accumulation of neuropeptides related to molting (1, 26–29). These biological processes reveal the existence of a complex and precise regulatory network at the premolt stage. To investigate the dynamic changes between the D0 and D2 substages, the transcriptomes of *dsGFP*-injected crabs at the D0 and D2 substages were compared. Global gene expression profiles of crabs at the D2 substage were distinct from crabs at the D0 substage, with 1,717 upregulated DEGs and 966 downregulated DEGs. Meanwhile, these downregulated DEGs related to transcription factor complex and RNA polymerase indicate that amounts of macromolecular complex are synthesized at the D0 substage to make sure the full preparation of proteins related to transcription factor that are needed for molting. Ribosomes are the important molecular machines that translate genetic information into proteins. The consistent trend of DEGs related to ribosome and transcription factor further supports the hypothesis that crab at the D0 substage is more active in preparing macromolecular complexes that are needed for molting. In *S. paramamosain*, the injection of *dsETH* with a dose of 5 µg/g could knock down *ETH* of crabs at the D0 substage but failed with crabs at the D2 substage (18). The expression level of *ETH* has a significant increase from the D0 substage to the D2 substage and then comes to a dramatic increase from the D2 substage to the E stage (18). It might be because the D0 substage is the preparatory phase of ETH mass synthesis, in which the synthesis of macromolecular complex that regulates the retaliatory increase of *ETH* is underway and has not been synthesized until the D2 substage. As an essential neuropeptide in the molting of *S. paramamosain*, the retaliatory regulatory mechanism may be a key for crab to live through the fragile stage under the changeable environment.

To investigate the correlative transcriptional pathways of *ETH* in *S. paramamosain* at the D0 substage, transcriptomes of the *dsETH-*injected group and the *dsGFP-*injected group were compared. It is interesting that knockdown of *ETH* leads to downregulation of genes enriched in carbohydrate metabolism, such as *TPI-like* and *TPI-A-like*, which are essential in glycolysis. Downregulation of *TPI* can lead to a decrease in the synthesis of ATP. Also, TPI deficiency is the most severe and lethal glycolytic enzymopathy (30, 31). We suggested that ETH may regulate the molting process by influencing glycolysis and energy supply at the D0 substage. Moreover, downregulated genes involved in one carbon pool by folate were screened, such as *MTHFR-like* and *FTCD-like*, of which the function of both is catalyzing NADH-dependent reduction of 5,10-methylenetetrahydrofolate to 5-methyltetrahydrofolate. It is worth mentioning that 5-methyltetrahydrofolate is essential to neural development, cell division, and embryogenesis (32, 33). As the whole molt cycle of crustacean is regulated by numerous neuropeptides released by nervous tissue, 5-methyltetrahydrofolate may affect the synthesis of neuropeptides by neural development. In contrast, transcriptional levels of peritrophins and hornerin-like involved in chitin binding were dramatically upregulated. In *Eriocheir sinensis*, chitinases involved in chitin binding showed a high expression level during the postmolt and ecdysis stages, and a very low level at the intermolt and premolt stages were detected (34), which indicated a potential role of chitin binding to molting. Meanwhile, researchers found that cuticular proteins analogous to peritrophins were abundantly distributed in the cuticle (35–37), and the knockdown of the genes can cause a delay of pupariation or mortality (38, 39). The relationship between *ETH* and *peritrophins* in *S. paramamosain* still needs more research.

At the D2 substage, transcriptomes of *dsETH*-injected crabs and *dsGFP*-injected crabs were compared. With the increase of *ETH* in crab, downregulated genes involved in calcium channel inhibitor activity were enriched, such as *FBC-L* and *FBC-L-like*. In mammals, FBC is localized to the primary cilium/basal body and plasma membrane in the renal epithelium and is thought to be a transcriptional target of hepatocyte nuclear factor 1β (40–42). Researchers found that a decline of *FBC* could result in abnormalities in ciliary morphology and possibly biliary cystogenesis in rat (43). The hepatopancreas is an important organ for the crab to store energy, substance, calcium, and so on. ETH may regulate molting by influencing the hepatopancreas through *FBC*. Moreover, upregulated genes were enriched in fat digestion and absorption, such as *AO*, *SPLA2*, and *SPLA2-like*, which are involved in the metabolism of linoleic acid, ether lipid, glycerophospholipid, and other lipids. AO is regarded as a biological regulator, especially for cell growth and differentiation (44). AO is also an important agent of pathological processes of rhabdomyolysis in rats (45). In mammals, the SPLA2 families are involved in a number of biological processes, such as modification of eicosanoid generation, inflammation, and host defense (46). In the study, ETH has a potential relationship with *AO* and *SPLA2s* in *S. paramamosain* at the D2 substage. We also observed the upregulation of several cardiac muscle contraction-related genes, such as *TPM1*, *MYH6*, and *MYH7*. TPM1 was first identified in skeletal muscles (Bailey) and plays a critical role in conjunction with troponin and the regulation of muscle contraction (47–49). Considering that muscle contraction is indispensable to shed the old cuticle for crab, ETH may trigger ecdysis through regulating *TPM1*.

## Conclusion

ETH has been determined to function in molting in several crustaceans; how ETH affects molting is still unclear in crustaceans. In the study, it is the first time to explore the potential downstream genes of ETH that are related to molting in crustaceans. Our results show that ETH has potential roles in carbohydrate metabolism, one carbon pool by folate, and chitin binding for crabs at the D0 substage, while with the process of molt cycle, the role of ETH turns to be involved in calcium channel inhibitor activity, fat digestion and absorption, and cardiac muscle contraction to facilitate the occurrence of molting. The findings of DEGs involved in the above pathways provide valuable insight into the role of ETH signaling in the regulation of crustacean molting.

## Data availability statement

The data presented in the study are deposited in the NCBI Sequence Read Archive (SRA) repository, and the sample information was registered in BioProject with accession number PRJNA813292.

## Author contributions

Conceptualization, S-FC and Y-FZ; methodology, S-FC, Y-FZ, C-MA, and C-GW; software, Y-FZ and Q-QW; validation, C-MA and WW; formal analysis, Y-FZ and WW; investigation, S-FC, Y-FZ, and WW; resources, C-GW; data curation, Y-FZ and Q-QW; writing—original draft preparation, S-FC, Q-QW, and Y-FZ; writing—review and editing, Q-QW and S-FC; visualization, Q-QW and C-MA; supervision, S-FC; project administration, S-FC; funding acquisition, S-FC. All authors have read and agreed to the published version of the manuscript.

## Funding

This research was funded by Guangdong Provisional Research Grant (#2014B020202014) and Guangxi Key Laboratory of Beibu Gulf Marine Biodiversity Conservation Fund (2019KB06).

## Conflict of interest

The authors declare that the research was conducted in the absence of any commercial or financial relationships that could be construed as a potential conflict of interest.

## Publisher’s note

All claims expressed in this article are solely those of the authors and do not necessarily represent those of their affiliated organizations, or those of the publisher, the editors and the reviewers. Any product that may be evaluated in this article, or claim that may be made by its manufacturer, is not guaranteed or endorsed by the publisher.
