# Supplementary figures and images for: Transcriptome responses of RNAi-mediated ETH knockdown in Scylla paramamosain at different premolt substages
Source: Front Endocrinol (Lausanne). 2022 Jul 28;13:917088. doi: 10.3389/fendo.2022.917088 (PMC9370559; doi:10.3389/fendo.2022.917088)

# Length Distribution

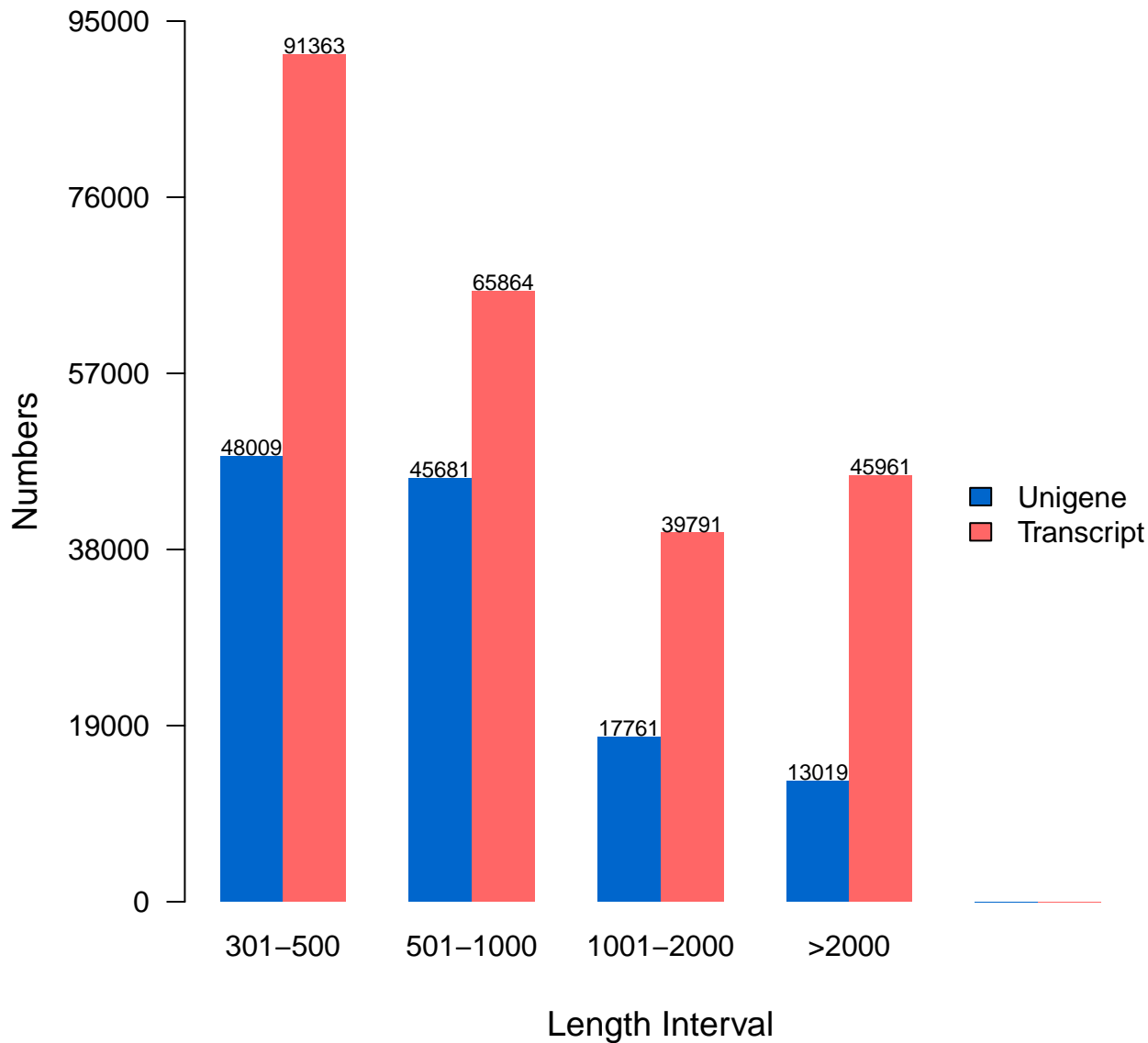

Supplement: Supplementary file 1 [file DataSheet_1.pdf]

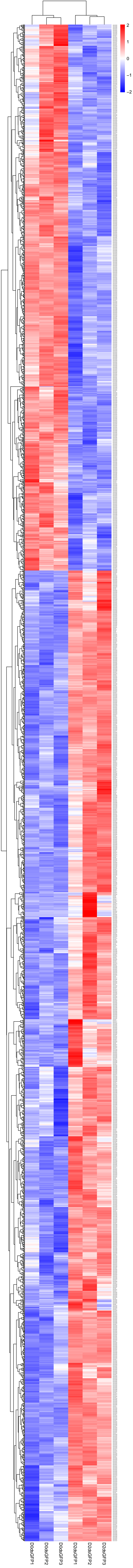

Supplement: Supplementary file 2 [file DataSheet_2.pdf]

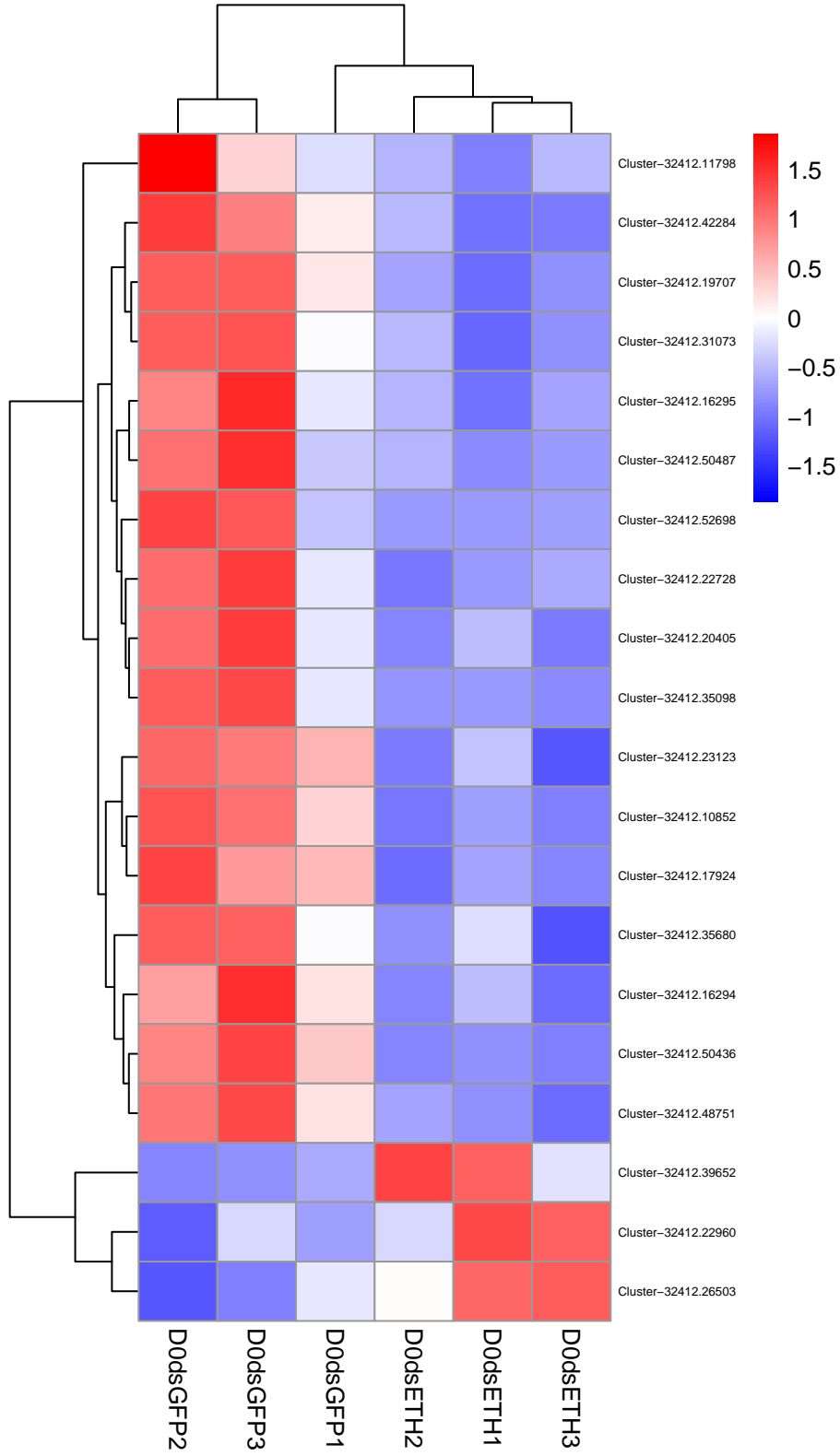

Supplement: Supplementary file 3 [file DataSheet_3.pdf]

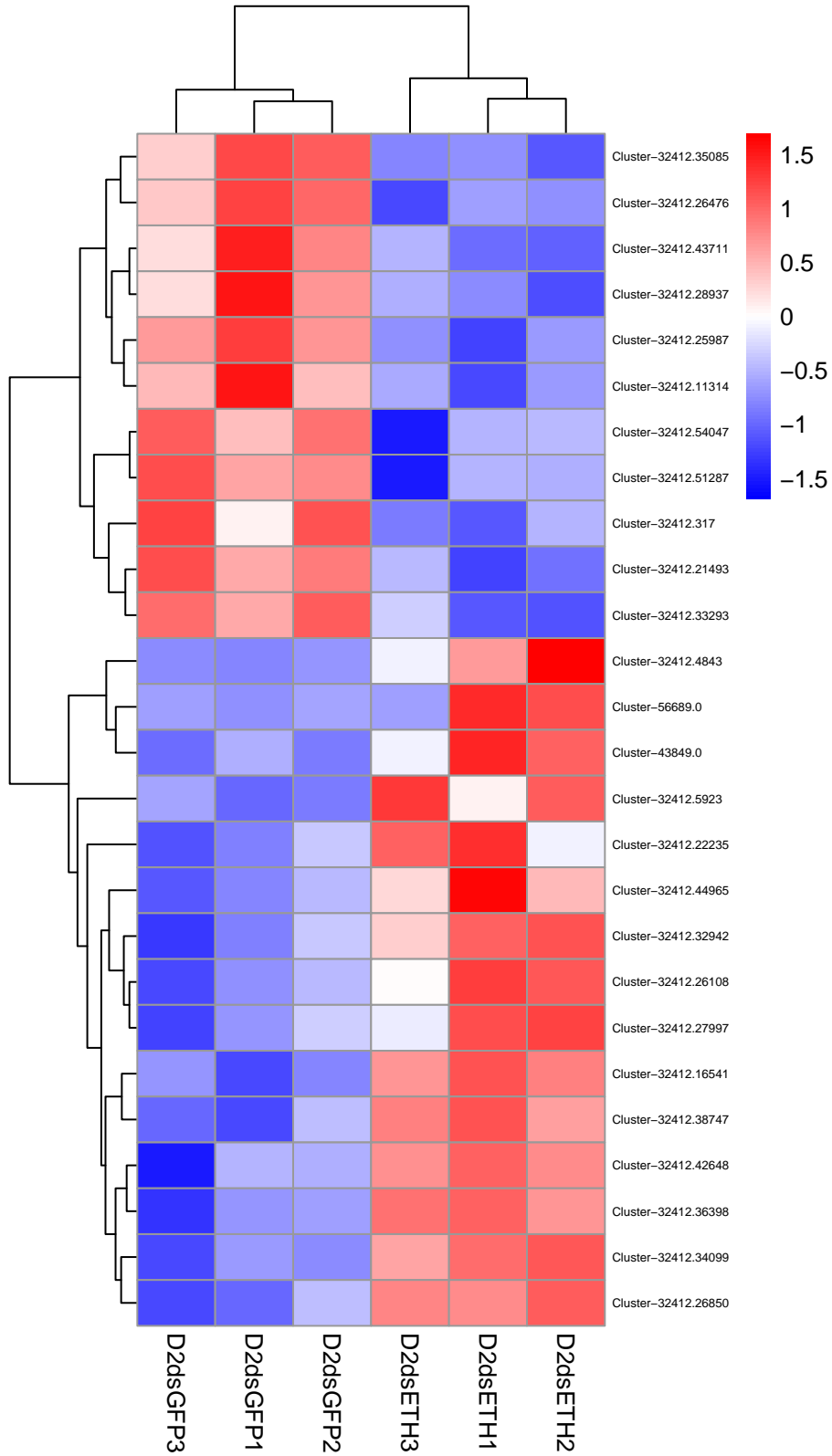

Supplement: Supplementary file 4 [file DataSheet_4.pdf]
