# Supplementary material for: Transcriptome responses of RNAi-mediated ETH knockdown in Scylla paramamosain at different premolt substages
Source: Front Endocrinol (Lausanne). 2022 Jul 28;13:917088. doi: 10.3389/fendo.2022.917088 (PMC9370559; doi:10.3389/fendo.2022.917088)
Supplement: Supplementary file 5 [file Table_1.docx]

**Table S1.** Primers used for dsRNA and PCR

| Primer name | Sequence (5′ to 3′) Primer name |
| --- | --- |
| *Sp18S-F* | ACTCAACACGGGGAACCTCAC |
| *Sp18S-R* | CAAATCGCTCCACCAACTAAG |
| *dsSpETH-F* | GGACTGTGCTGGCTGTGGTG |
| *dsSpETH-R* | GGCGATCTGAGGCAAAGGT |
| *T7SpETH-F* | TAATACGACTCACTATAGGGGACTGTGCTGGCTGTGGTG |
| *T7SpETH-R* | TAATACGACTCACTATAGGGGCGATCTGAGGCAAAGGT |
| *RT-SpETH-F* | CGATGCTCTCTGTTCTGGACTCA |
| *RT-SpETH-R* | CGCTCGAGTCTCACTTCTGCA |
| *qSpETH-F* | GAATGGCGTGGTTGGTG |
| *qSpETH-R* | GGGCGTCCTCCTCTGATA |
| *qSpTIM-like-F* | ATTGGAGGGCACTTTATCG |
| *qSpTIM-like-R* | GCTCAGCGCCAGTATCG |
| *qSpTIM-A-like-F* | GCACGGCAAGCAACAAG |
| *qSpTIM-A-like-R* | TTCACGGTGGCACGATT |
| *qSpMTHFR-like-F* | AACAGTGGTCAGTGCGTGAA |
| *qSpMTHFR-like-R* | TACCCTGTACTTACTCCAAACCTTA |
| *qSpFTCD-like-F* | GAACTTCCTGACGCCTGTGAC |
| *qSpFTCD-like-R* | TACGAGGGCCTGAAGGATAAG |
| *qSpperitrophin-like 1-F* | ACTACTGTATGGACGCTGGAC |
| *qSpperitrophin-like 1-R* | GGACACTTAGGGAAGAAACC |
| *qSpperitrophin-like 2-F* | TGAGGAACAGGCATCGG |
| *qSpperitrophin-like 2-R* | ACGGAGACCAGGGCAAG |
| *qSpperitrophin-1-like-F* | CAGGAAGGGTGTCTGCTCG |
| *qSpperitrophin-1-like-R* | TGGTGGCTCAATGATGACG |
| *qSpperitrophin-44-like-F* | TCCAGTCCTCCTCCAGC |
| *qSpperitrophin-44-like-R* | GACGCAGTCCGAATCAGT |
| *qSphornerin-like-F* | CACGAGTCCCTCCGAACA |
| *qSphornerin-like-R* | ACACCCGTCCCAACCA |
| *qSpFBC-L-F* | CTGTCACGCCCTCTACTGATG |
| *qSpFBC-L-R* | CAAGTGCTGGGACGATGC |
| *qSpFBC-L-like-F* | GGATAACTGTGAGGACGCTGAT |
| *qSpFBC-L-like-R* | CATAATAGTGGATGCCTTGCTG |
| *qSpAOD-F* | GGAGGTGGAGTCGCTGTA |
| *qSpAOD-R* | TGGCCTGTGGCGGAAT |
| *qSpSPLA2-F* | TGACGCCCTTTGAATACC |
| *qSpSPLA2-R* | GGGTGCCATCTACTACACTA |
| *qSpSPLA2-like-F* | ACCTCGGAGACTCACCAACA |
| *qSpSPLA2-like-R* | AAGCCGCAGTAGCACCC |
| *qSpTPM1-F* | GGTGGCGGAAGAGCAGA |
| *qSpTPM1-R* | CGACAGGCTTGAAGACGA |
| *qSpMYH6-F* | AAGAGGCTGTCGCTGATC |
| *qSpMYH6-R* | CCTGGCGTTCGTGTTC |
| *qSpMYH7-F* | AGGTTACTGTGCCTTCCAT |
| *qSpMYH7-R* | CAGCCTCAGTGCCATCA |
